# Metabolically healthy obesity (MHO) and factors influencing its prevalence in a population in North-West Lahore

**DOI:** 10.12669/pjms.41.5.11694

**Published:** 2025-05

**Authors:** Mohammad Perwaiz Iqbal, Tehreem Sajjad, Saleem Perwaiz Iqbal, Muhammad Khurram

**Affiliations:** 1 Prof. Mohammad Perwaiz Iqbal, Department of Biological and Biomedical Sciences, Aga Khan University, Karachi, Pakistan. Department of Life Sciences, University of Management and Technology, Lahore, Pakistan; 2 Tehreem Sajjad Department of Life Sciences, University of Management and Technology, Lahore, Pakistan; 3 Saleem Perwaiz Iqbal Department of Community Medicine, Shalamar Medical and Dental College, Lahore, Pakistan; 4 Muhammad Khurram Department of Life Sciences, University of Management and Technology, Lahore, Pakistan

**Keywords:** Abdominal obesity, Dietary habits, Lifestyle factors, Metabolic abnormalities, Metabolically healthy obesity, Metabolically unhealthy obesity

## Abstract

**Objective:**

To investigate the prevalence of metabolically healthy obesity (MHO) in a population residing in Northwest of Lahore and study the association of some lifestyle factors influencing it.

**Methods:**

In a cross-sectional design, a study was conducted at the Department of Life Sciences, University of Management and Technology, Lahore, in which 496 adults of either gender having abdominal obesity were included with informed consent. Those having none or one of the following metabolic abnormalities – hypertension, hypertriglyceridemia, hyperglycemia and low HDL-cholesterol along with abdominal obesity were classified as having metabolically healthy obesity (MHO). These metabolic abnormalities were measured in all recruited subjects. A questionnaire comprising demographic and lifestyle habits was used for information about factors influencing MHO. Chi-square test and logistic regression were used for studying the association of these factors with MHO. The duration of the study was from August 2022 to October 2023.

**Results:**

Prevalence of MHO was 11% of the population studied. It was inversely related to age. Regarding association of MHO, compared to males, the adjusted odds ratio (AOR) among females was 74% lower [AOR = 0.26; 95% CI (0.11-0.59)]. Moreover, married individuals had 0.36 times odds of MHO compared to single ones [AOR = 0.36; 95% CI (0.16-0.8)]. Skipping breakfast less than once a week had 0.11 times odds of MHO compared to those who were skipping breakfast everyday [AOR = 0.11; 95% CI (0.02-0.49)].

**Conclusion:**

Eleven percent prevalence of MHO suggests the need to create awareness amongst communities for adoption of healthy lifestyle habits for better metabolic health.

## INTRODUCTION

In the Western population, there are many people who are fat but metabolically fit and for them a term has been coined as having metabolically healthy obesity (MHO).^1^ Such people are obese but according to recent definition of MHO have none or at the most one of the following four components of metabolic syndrome (MetS) which include hyperglycemia (fasting), low HDL-cholesterol, hypertension and hypertriglyceridemia.^2^,^3^ It is normally also described as absence of any metabolic disorder or cardiac disease such as dyslipidemia, Type-2 diabetes, hypertension, and atherosclerosis in a person having obesity.^4^

Central obesity as per WHO’s South Asia specific cut-offs, is a growing public health problem in Pakistan with prevalence as high as 73.1% among adults.^4^,^5^ With increasing urbanization, the younger population is getting more prone to developing obesity and diabetes because of changing dietary habits and a sedentary lifestyle.^4^,^6^ This accumulation of fat in and around organs rather than skin is more injurious to health as it makes body more prone to developing metabolic abnormalities.^6^,^7^

A number of studies have been carried out on prevalence of MHO in various countries such as USA, United Kingdom, rural areas of China and Spain showing percentages ranging from 6.5% to 68%. In the USA, the prevalence of MHO was found to be 54% and 24% among adults in the age groups 19-44 years and 45-85 years, respectively.^8^ In United Kingdom, overall prevalence of MHO was 34% among adults.^9^ Zhang et al. reported an overall prevalence of MHO in rural China to be 23.1%.^10^ In Spain, prevalence of MHO was 6.4% among adults.^11^ Except for a study carried out in Kuwait by Oguoma et al. on Arab population and South Indian population including Pakistanis residing over there to determine the prevalence of MHO, there is no study carried out in Pakistan to find out the prevalence of MHO in Pakistani population.^12^ Therefore, we embarked on this study to find out the prevalence of MHO and metabolically unhealthy obesity (MUHO) in a population in North-West of Lahore.

We used the International Diabetes Federation (IDF) criteria for South and South-East Asians for diagnosis of the following cardiometabolic abnormalities.^13^

- Waist circumference: males ≥ 90 cm; female ≥ 80cm.
- Blood pressure: systolic BP ≥130 mmHg and diastolic BP ≥ 85 mmHg.
- Fasting blood glucose: ≥ 100 mg/dL (or its equivalent).
- Triglycerides: ≥ 150 mg/dL (or its equivalent).
- High density lipoprotein-cholesterol (HDL-C): Males < 40 mg/dL (or its equivalent); Females < 50 mg/dL (or its equivalent).

The presence of none or at the most one of the above metabolic abnormalities along with abdominal obesity was used to call the subject as having MHO.

## METHODS

A cross-sectional study was conducted from August 7, 2022, to October 25, 2023 on North-Western population of Lahore, at the Department of Life Sciences, University of Management and Technology (UMT), Lahore. Four hundred and ninety six obese adults (age range: 18-70 years) were recruited with informed consent. Blood samples were carefully withdrawn with the help of a phlebotomist after an eight hour fast. Serum was analyzed for biomarkers such as glucose, triglycerides and HDL-cholesterol. Serum was separated immediately to prevent glycolysis. Glucose is stable in serum up to 8 hours at 25°C or up to 72 hours at 4°C.

Considering 38% prevalence of MHO in Kuwait from a previous study by Oguoma et al. and his associates, our sample size was estimated to be 358 at 5% precision and at 95% confidence interval.^12^ We recruited 496 subjects from the North-West of Lahore, who fulfilled the selection criteria of this research.

### Ethical Approval:

The study had been approved by the Biosafety and Bioethics Committee (Ref: SBBC-2023-09; dated August 7, 2023), University of Management and Technology, Lahore.

### Inclusion & Exclusion Criteria:

Regarding inclusion criteria, both males and females, apparently healthy adults, age between 18-70 years and having abdominal obesity according to the WHO criteria for South Asians were included in the study, while for exclusion criteria, pregnant females and subjects having a chronic disease (except diabetes and hypertension) such as cancer, liver disease, kidney disease, malabsorption syndrome were excluded from the study.

There is no consensus among the scientific community about the precise definition of MHO, however a vast majority of them agree that it refers to absence of metabolic abnormalities such as high fasting serum glucose, dyslipidemia, and hypertension in obese subjects.^14^ In the present study, we used the criteria of MHO as having zero or one metabolic abnormality along with obesity.^8^,^15^,^16^ In other words, the obese subjects had none or one of the following cardiometabolic risk factors (fasting hyperglycemia, hypertriglyceridemia, low HDL-cholesterol and high blood pressure). For the determination of abdominal obesity, WHO criteria for central obesity for South Asians was used. It refers to the waist circumference ≥ 80 cm in women and ≥ 90 cm in men.^5^

All study participants were subjected to physical and clinical examination and determination of waist circumference (cm), blood pressure (mmHg), fasting serum glucose level (mg/dL), serum triglycerides (mg/dL) and serum HDL-cholesterol (mg/dL). Demographic and clinical characteristics of recruited subjects were also determined such as gender, age, history of diabetes, heart disease and hypertension.

Serum levels of glucose, triglycerides and HDL cholesterol were determined using commercially obtained kits and following manufacturers’ instructions. HDL and triglyceride kits were obtained from Bioactive Diagnostic Systems, Voehl, Germany, while kit for serum glucose was purchased from ARENA Bioscience, Ismailia, Egypt. The equipment for analyzing these kits was MicroLab 300, a semi-automated Biochemistry Analyzer (Merck, Germany).

There is evidence that certain dietary and lifestyle habits do influence the risk of metabolic abnormalities. For example, Iqbal and his associates have reported that several dietary and lifestyle habits could increase the risk of metabolic syndrome.^17^ Some of these dietary and lifestyle factors were included in the questionnaire for the study subjects. Briefly, these included smoking status, television viewing time, frequency of consumption of tea/coffee, soft-drinks, duration of sleep, frequencies of dining out, skipping breakfast, taking late dinners and meal finishing time.

### Statistical Analysis:

Epidata version 3.1 software was used for data entry and documentation. For statistical analysis, data were extracted from Epidata and entered in Statistical Package for Social Sciences (SPSS) version 23. Percentages and frequencies were calculated to find significant variables in different categories. For continuous variable, mean ± standard deviation (SD) was obtained. Chi-square was applied to find out statistical significance. Logistic regression was applied using significant variables while adjusting for co-variates. The significant p-value for every analysis was less than 0.05.

## RESULTS

Mean ± SD for age of the recruited subjects was found to be 40 ± 10 years. Table-IA shows comparison of sociodemographic factors between MHO and MUHO groups in this population. Compared to males, significantly more females belonged to MUHO group (p-value = 0.04). A considerable difference was observed in various categories of marital status between MHO group and MUHO group (p-value <0.01). Greater proportions of subjects with relatively better education (intermediate and above) were found in MUHO group compared to MHO group (p-value=0.04). Significant differences were observed in various categories of occupation between the MHO group and MUHO group (p-value = 0.01). Monthly household income, however, was not significantly different between MHO group and MUHO group in various categories of income (p-value = 0.72). The comparison of sociodemographic characteristics between MHO and MUHO was analyzed using Chi Square test. In order to find out the age-related trends in prevalence of MHO and MUHO the recruited subjects were divided into three groups – 18-40 years, 41-60 years and above 60 years of age. Table IB shows that 78.2% of subjects in the 18–40-year age group had MHO, while in the 41–60-year age group, there were 21.8% metabolically healthy obese, This indicates that young adults in this population have a high prevalence of MHO and low prevalence of MUHO.

**Table-IA:** Comparison of percentages of sociodemographic factors between metabolically healthy obesity (MHO) and metabolically unhealthy obesity (MUHO) groups (n=496) [n (%)].

Sociodemographic characteristics		MHO (n=55)	MUHO (n=441)	χ^2^	p-value
Sex	Male	29 (15)	170 (85)	4.09	0.04
	Female	26 (9)	271 (91)		
Marital status	Single	30 (23)	100 (77)	26.70	< 0.01
	Married	21 (8)	248 (92)		
	Widowed	4 (7)	53 (93)		
	Divorced/separated	0 (0)	40 (100)		
Education	None	0 (0)	6 (100)	10.85	0.04
	Primary	9 (21)	33 (79)		
	Middle	13 (17)	63 (83)		
	Secondary	11 (13)	73 (87)		
	Intermediate/diploma	12 (8)	143 (92)		
	Graduate	8 (7)	114 (93)		
	Professional degree	2 (18)	9 (82)		
Occupation	Unemployed	9 (18)	40 (82)	13.30	0.01
	Unskilled worker	15 (18)	70 (82)		
	Skilled worker	16 (10)	139 (90)		
	Clerical/shop/farm	10 (6)	165 (94)		
	Professional (white collar)	5 (24)	16 (76)		
	Retired	0 (0)	11 (100)		
Average HH income	< PKR 30000	22 (13)	143 (87)	1.32	0.72
	PKR 30001-50000	20 (10)	175 (90)		
	PKR 50001-80000	11 (9)	106 (91)		
	> PKR 80000	2(10)	17 (90)		

**Table-IB:** Association of age categories with the presence of MHO among the sampled population (n=496) [n (%)]

Age-based categories		MHO (n=55)	MUHO (n=441)	χ^2^	p-value
Age (in years)	18-40 years	43 (78.2)	159 (36.1)	36.20	< 0.01
	41-60 years	12 (21.8)	268 (60.8)		
	> 60 years	0 (0)	14 (3.2)		

Table-II indicates the comparison of lifestyle habits between MHO and MUHO groups. Finishing meals in less than 20-minutes time or more than 20 minutes appeared to have little influence in both MHO group and MUHO group as there was no statistically significant difference between the two groups (p-value = 0.09). Similarly, the frequency of taking dinner after 10 pm and earlier than that had no significant effect between the two groups (p-value = 0.38). Frequencies of skipping breakfast (frequently vs. less frequently) were significantly different among the MHO and MUHO groups (p-value = 0.01). Frequencies of dining out between the two groups were not statistically significant (p-value = 0.93). Similarly, various sleep durations were not considerably varying between the two groups (p-value = 0.54). Frequencies of smokers and non-smokers were significantly different among the MHO and MUHO groups (p-value = 0.03).

**Table-II:** Comparison of percentages of lifestyle habits between metabolically healthy obesity (MHO) and metabolically unhealthy obesity (MUHO) groups (n=496) [n (%)].

Lifestyle habits		MHO (n=55)	MUHO (n=441)	χ^2^	p-value
How fast do you finish your meal?	More than 20 minutes	7 (10)	60 (90)	3.86	0.09
	10-20 minutes	30 (15)	175 (85)		
	less than 10 minutes	18 (8)	206 (92)		
How frequently do you eat after 10 pm?	Everyday	7 (18)	31 (82)	1.74	0.38
	3-6 times/wk	16 (12)	114 (88)		
	1-2 times/wk	15 (19)	156 (91)		
	Less than once a week	11 (9)	106 (91)		
	Never/rare	6 (15)	34 (85)		
How frequently do you skip breakfast?	Everyday	5 (31)	11 (69)	9.16	0.01
	3-6 times/wk	22 (16)	117 (84)		
	1-2 times/wk	9 (6)	131 (94)		
	Less than once a week	9 (7)	114 (93)		
	Never/rare	10 (13)	68 (87)		
How frequently do you dine out?	Everyday	0 (0)	6 (100)	0.05	0.93
	3-6 times/wk	11 (11)	88 (89)		
	1-2 times/wk	14 (12)	108 (88)		
	Less than once a week	19 (12)	145 (88)		
	Never/rare	11 (10)	94 (90)		
How many hours do you sleep in a day?	< 6 hrs/day	5 (10)	44 (90)	1.23	0.54
	6-8 hrs/day	28 (13)	190 (87)		
	> 8 hrs/day	22 (10)	207 (90)		
Smoking status	None	43 (10)	380 (90)	11.82	0.03
	Past	4 (8)	44 (92)		
	Current	8 (32)	17 (68)		
Smokeless tobacco consumption	None	47 (11)	395 (89)	1.40	0.49
	Past	5 (18)	23 (82)		
	Current	3 (12)	23 (88)		
Television viewing	less than 1 hr	9 (12)	65 (88)	0.34	0.95
	1-2 hrs	28 (12)	214 (88)		
	2-3 hrs	10 (10)	90 (90)		
	more than 4 hrs	8 (10)	72 (90)		
How frequently do you drink soft drinks (coke, sprite, cola)?	Everyday	5 (11)	39 (89)	0.70	0.56
	4-6 times a week	32 (12)	232 (88)		
	1-2 times a week	9 (8)	110 (92)		
	less than once a week	9 (13)	60 (87)		
How frequently do you drink tea/coffee?	more than 3 times a day	6 (7)	77 (93)	1.51	0.15
	1-2 times a day	39 (13)	253 (87)		
	none/rare	10 (8)	111 (92)		

However, consumption of smokeless tobacco had no significant effect among the two groups. Similarly, the duration of television viewing, frequency of taking soft drinks every day and frequency of drinking tea/coffee every day had no significant effect among MHO and MUHO groups (p-value = 0.95, 0.56, 0.15, respectively). Chi Square test was employed for comparison.

Table-III shows the adjusted odds ratio (with 95% CI) of characteristics associated with MHO. In comparison to males, the adjusted odds ratio (AOR) was 0.26 times among females [AOR = 0.26; 95% CI (0.11 – 0.59)]. Regarding marital status, compared to those who remained single, the married ones were having 0.36 times the odds of MHO [AOR = 0.36; 95% CI (0.16 – 0.80)]. Education level appeared to have no influence on the development of MHO. Similarly, occupation, finishing mealtimes, smoking status and consumption of tea/coffee did not seem to be related to MHO in this population. Regarding skipping breakfast, compared to those who were skipping breakfast every day, those who had the habit of skipping breakfast less frequently (one times per week) had 0.11 times the odds of having MHO [AOR = 0.11; 95% CI (0.02 – 0.49)].

**Table-III:** Adjusted odds ratio (with 95% CI) of characteristics associated with MHO (n=496).

Parameters		Adjusted OR*	95% CI	
			Lower	Upper
Age (years)		0.90	0.86	0.95
Gender	Male	1.00		
	Female	0.26	0.11	0.59
Marital status	Single	1.00		
	Married	0.36	0.16	0.80
	Widowed/divorced	0.41	0.10	1.63
Education	None & Primary	1.00		
	Middle	1.34	0.40	4.43
	Secondary	0.91	0.26	3.24
	Intermediate/diploma	0.55	0.16	1.88
	Graduate	0.39	0.10	1.58
	Professional degree	0.78	0.05	11.05
Occupation	Unemployed/retired	1.00		
	Unskilled worker	1.05	0.34	3.19
	Skilled worker	1.09	0.36	3.35
	Clerical/shop/farm	0.56	0.17	1.90
	Professional (white collar)	2.97	0.41	21.57
How fast do you finish your meal?	More than 20 minutes	1.00		
	10-20 minutes	2.83	0.93	8.63
	less than 10 minutes	1.74	0.54	5.66
How frequently do you skip breakfast?	Everyday	1.00		
	3-6 times/wk	0.29	0.07	1.17
	1-2 times/wk	0.12	0.03	0.56
	Less than once a week	0.11	0.02	0.49
	Never/rare	0.23	0.05	1.06
Smoking status	None	1.00		
	Past	0.43	0.11	1.69
	Current	2.89	0.84	9.89
How frequently do you drink tea/coffee?	more than 3 times a day	1.00		
	1-2 times a day	1.92	0.68	5.41
	none/rare	1.04	0.30	3.59

* Adjusted for significant co-variates.

## DISCUSSION

In this study the objective was to find out the prevalence of MHO and MUHO in Northwestern population of Lahore. This information was important for public health professionals who are concerned about the rising problem of abdominal obesity in South Asia.^18^ Results showed that in Lahore population, prevalence of MHO was quite low (11%) compared to 37.9% prevalence among South Asians (including Pakistanis) in Kuwait on the basis of abdominal obesity.^12^ The difference in terms of prevalence of MHO among Pakistan population and South Asians could be due to lifestyle and dietary habits between the two groups. Moreover, logistic regression analysis of our data revealed that compared to males the adjusted odds of having MHO was 0.26 times among females in Lahore population [AOR = 0.26; 95% CI (0.11-0.59)] indicating that females are more likely to be unhealthy obese in Lahore population compared to males. This is in line with the results obtained by Oguoma and his associates on South Asian population in Kuwait reporting MHO percentage to be lower in females compared to males.^12^ In a study carried out on South Indian population in Chennai, the prevalence of MHO was found to be 13.3% using the obesity cut-off of ≥ 25 Kg/m^2^).^19^ However, the mean age of the population was lower than the mean age of Lahore population (36 ± 10 years vs. 40 ± 10 years), suggesting that the prevalence of MHO in Southern Indian population would have been lower than their reported value, had they included subjects with higher ages. In a recent report from Iran by Taherifard and his associates in which age-and gender-standardized prevalence of MHO was found to be 2.3%.^20^ Another important result of the present study was that compared to those who remained single, married subjects were less likely to be metabolically healthy obese.

Several factors may be contributing to the relatively low percentage of MHO among Lahore population, compared to the South-Asians living in Kuwait. For example, high stress levels associated with city life could lead to increased prevalence of hypertension. Additionally, the frequent utilization of various sugar-rich food items such as soft drinks, cakes, sweets and ice cream can lead to increased risk of hyperglycemia. Moreover, consumption of oil and fat-rich traditional Lahori foods (especially in breakfast) such as “halwa puri”, “nihari” and “siri-paya” is likely to increase the chances of hypertriglyceridemia leading to abdominal obesity. This is supported by the observation in the present study that those skipping breakfast less frequently (1-2 times per week) had 0.11 times odds of having MHO [AOR=0.11; 95% CI (0.02-0.49)].

There have been several studies showing that the prevalence of MHO is inversely related to age. For example, Camhi et al. have shown that the prevalence of MHO decreases with increasing age: 68%, 54% and 24% in adolescents, in adults with age19-44 years and older subjects in age group 45-85 years, respectively.^8^ We also found the inverse relationship between age and MHO. We included some of the dietary and lifestyle factors as used by Iqbal et al. in their study on Malaysian population which could influence the odds of having metabolic abnormalities.^17^ However, except for skipping breakfast less frequently, no other dietary and lifestyle habit was found to be associated with MHO. The fact that female population of Northwest of Lahore was found to be more vulnerable to the risk of developing MUHO may be regarded as a strength of this study as it is likely to alert the public health professionals to focus more on this vulnerable group.

Middle-aged women, especially after menopause generally have a sedentary lifestyle in an urban city like Lahore, compared to women in rural areas. Compared to men, women have fewer opportunities to go to healthcare facilities for their metabolic health. This could be one of the reasons for higher prevalence of MUHO among females in the studied population.

### Limitations:

The present study had a few limitations as well. Ooi et al. in a multiethnic population have shown younger age, absence of paternal obesity and ethnicity as independent predictors of MHO.^21^ We could not include in the questionnaire paternal obesity and racial background of the recruited subjects. Therefore, absence of information about these parameters could be a limitation of the present study. In addition, we could not obtain information about the engagement in physical activity, which is a well-known predictor of metabolic health.^22^ Moreover, the sampling was done using a convenient sampling design and was confined only to the Northwestern region of Lahore. Therefore, the findings may not be generalizable to the entire population of Lahore. Although MHO is a transitory phase in the life of an obese person, it points towards the metabolic health of an obese population.^1^,^15^ It enables identification of more vulnerable groups in obese population so that timely intervention could delay this transition from MHO to MUHO. The plausible mechanism by which the transition of MHO to MUHO could take place has been delineated. It is stated that obesity being a low-grade inflammatory state often leads to cardiometabolic abnormalities.^22^ However, those with unhealthy lifestyle habits would be more vulnerable. The challenge before the public health personnel is to delay this transition as much as possible in the vulnerable population through adoption of healthy lifestyle. It is recommended that public awareness campaigns through mass media should be started along with the initiation of community outreach programs to enlighten the population in Lahore to adopt healthier food and lifestyle choices for prevention of obesity and metabolic abnormalities, especially among the high-risk groups such as women and elderly subjects to reduce the rising burden of abdominal obesity and cardiometabolic risks in this urban population in Lahore.

## CONCLUSION

Eleven percent MHO was found in an obese urban population in Northwest Lahore, indicating that a vast majority of them were metabolically unhealthy. Women were more affected compared to males. Awareness campaigns are needed for the adoption of heathy food and lifestyle habits to reduce the burden of obesity and other metabolic abnormalities in this urban population.
